# Контрацепция у подростков с ожирением и сахарным диабетом

**DOI:** 10.14341/probl12760

**Published:** 2022-12-20

**Authors:** М. Р. Шайдуллина, Ф. В. Валеева, А. Ф. Субханкулова, П. А. Хусиева

**Affiliations:** Казанский государственный медицинский университет; Детская республиканская клиническая больница; Казанский государственный медицинский университет; Казанский государственный медицинский университет; ГАУЗ «Детская республиканская клиническая больница» Минздрава Республики Татарстан

**Keywords:** контрацепция, подростки, сахарный диабет, безопасность, эффективность

## Abstract

В современных условиях большинство подростков имеют сексуальный дебют в возрасте 15–19 лет. При этом при первом половом контакте какими-либо методами контрацепции пользовались только 44% девушек и молодых женщин в возрасте 15–24 лет. Принятие решения в случае наступления беременности в подростковом возрасте — сложный выбор, любой вариант развития событий чреват серьезными медицинскими и социальными проблемами. Осложнения после искусственного прерывания беременности оказывают негативное влияние на фертильность девушки в будущем. А инсулинозависимый сахарный диабет и артериальная гипертензия, являющиеся наряду с ожирением одними из компонентов метаболического синдрома, отнесены Всемирной организацией здравоохранения (ВОЗ) к числу заболеваний, подвергающих женщину повышенному риску в случае незапланированной беременности. В статье рассматриваются проблемы взаимодействия врача с девушкой-подростком, наблюдающейся с данными эндокринопатиями, при обсуждении ее сексуального здоровья, представлен анализ литературы, отражающей возможные влияния средств контрацепции на течение основной патологии. Оценка рисков и преференций от применения того или иного средства контроля фертильности позволила авторам сформировать перечень препаратов, допустимых к использованию при сахарном диабете и ожирении. В работе содержатся сведения о процедуре старта контрацепции, особенностях дальнейшего динамического наблюдения за пациентом.

## Abstract (en)

Today most adolescents have their first sexual experience at the age of 15–19. However, only 44% of girls and young women (15–24 years old) report about contraception at that moment. A decision on pregnancy in adolescence is a difficult choice and any scenario may cause serious medical and social problems. Complications after an artificial abortion have a negative impact on a woman’s fertility. Diabetes mellitus type 1 and arterial hypertension accompanied with obesity within the metabolic syndrome are defined by the World Health Organization (WHO) as diseases, which increase risk of an unplanned pregnancy. The article consoders problems of interaction of a doctor and a teenage girl with endocrinopathy, when discussing her sexual health, the analysis of the literature reflecting the influence of contraception on the course of the underlying pathology is presented. The authors formed a list of drugs acceptable for use in diabetes and obesity based on assessment of risks and preferences from the use of different methods of fertility control. The work contains information about the procedure of starting contraception, the rules of future dynamic monitoring of the patient.

В современных условиях большинство подростков имеют сексуальный дебют в возрасте 15–19 лет [1]. Однако только 44% девушек и молодых женщин в возрасте 15–24 лет использовали какие-либо методы контрацепции при первом половом контакте [2]. Принятие решения в случае наступления беременности в подростковом возрасте — сложный выбор, любой вариант развития событий чреват серьезными медицинскими и социальными проблемами. Осложнения после искусственного прерывания беременности оказывают негативное влияние на фертильность женщины в будущем. А инсулинозависимый сахарный диабет и артериальная гипертензия, являющаяся наряду с ожирением одним из компонентов метаболического синдрома, отнесены ВОЗ к числу заболеваний, подвергающих женщину повышенному риску в случае незапланированной беременности [3]. Использование контрацепции ограничивают следующие причины: в 19,8% случаев это отсутствие информации о ее методах и в 14,9% — недоступность квалифицированной консультации по их выбору [4].

Работа врача с подростками при обсуждении их репродуктивного здоровья и сексуальной жизни имеет свои юридические и этические нюансы. Согласно российскому законодательству, возраст половой зрелости составляет 16 лет, и половые отношения между лицом, не достигшим таковой, и человеком 18 лет и старше могут преследоваться по закону [5]. В то же время молодые люди старше 15 лет имеют право принимать собственные решения в отношении своего здоровья независимо от желания их родственников или законного представителя, врач должен обеспечить конфиденциальность полученной от подростка информации и соблюдение врачебной тайны [6]. Однако психологические особенности этого возраста, недостаточная социальная зрелость могут негативно сказаться на принятии важных решений.

Обсуждение проблем сексуального здоровья подростка с сахарным диабетом требует от врача дополнительной информированности о возможных лекарственных взаимодействиях контрацептивных и сахароснижающих средств, влиянии препаратов половых стероидов на риски формирования поздних осложнений основной патологии. The Intеrnational Society of Pеdiatric and Adolеscent Diabetes (ISPAD) и American Diabetes Association (ADA) рекомендуют начинать разговор о репродуктивном здоровье с подростком, наблюдающимся по поводу сахарного диабета, в начале пубертатного периода, даже до старта менструальной функции [7]. Изменение композиционного состава тела, увеличение объема циркулирующей крови при ожирении также имеют принципиальное значение для фармакокинетики лекарственных препаратов, что нельзя не учитывать при выборе средства контрацепции у этой категории пациентов. При наличии ожирения у женщины встает вопрос не только о надежности контрацептива, но и о его влиянии на метаболические процессы в организме и систему гемостаза. Очень часто специалистом, которому подростки с сахарным диабетом или ожирением впервые задают свои вопросы об интимной стороне жизни, становится детский эндокринолог, уже установивший доверительные отношения с пациентом в процессе достижения оптимального метаболического контроля.

Уварова Е.В. [8] предлагает алгоритм взаимодействия с девочкой-подростком, ищущей информации о возможных методах контрацепции, показавшийся нам достаточно рациональным и применимым в том числе и при общении с пациентами с сахарным диабетом или ожирением.

Шаг 1. Убеждение и здравый смысл — предупреждение отчаянных, необдуманных поступков. Данный этап требует доверительной атмосферы, позволяющей девушке открыто говорить об интимной стороне своей жизни. Это время для обсуждения необходимости (пациентке менее 15 лет) или возможности (пациентка старше 15 лет) участия в разговоре родителей (подростки были более привержены выбору высоконадежной контрацепции, если их мать была информирована о сексуальных контактах дочери) [8]. Нельзя замалчивать возможность развития побочных эффектов контрацептивов, однако акцент должен быть сделан на несомненном преимуществе их при грамотном использовании. Следует отметить, что в дополнение к профилактике незапланированной беременности контрацепция поможет подростку контролировать такие медицинские проблемы, как различные варианты нарушения менструального цикла и акне [9].

На наш взгляд, крайне важным моментом является обсуждение нежелательных последствий для здоровья как матери, так и будущего ребенка незапланированной беременности при сахарном диабете на фоне отсутствия должного гликемического контроля — инфекции, преэклампсия, прерывание беременности на любом сроке, мертворождение, гипоксия плода, дистресс-синдром, грубые пороки развития [10] — с одной стороны, и возможность вынашивания и рождения здорового потомства в случае адекватной прегравидарной подготовки и строгого метаболического контроля — с другой [10][11].

Перед обсуждением предпочтительных для подростка методов контрацепции необходимо убедиться в отсутствии нежелательной беременности, если такой уверенности нет, необходимо провести тест и обговорить возможность применения методов экстренной контрацепции [12].

Средства экстренной контрацепции не имеют абсолютных противопоказаний [3]. К наиболее доступным из них относятся: левоноргестрел (1,5 мг для перорального применения однократно), улипристала ацетат1 (30 мг перорально однократно), мифепристон (10 мг внутрь однократно), введение Cu-ВМС (негормональные внутриматочные средства с медью), отличающихся наименьшей эффективностью по сравнению с перечисленными выше. Наибольшей эффективностью обладают Cu-ВМС, которые могут быть установлены сразу после получения отрицательного результата теста на беременность в течение 5 дней после незащищенного полового акта или в течение 5 дней после дня наиболее ранней ожидаемой овуляции независимо от количества незащищенных половых актов [13]. Улипристала ацетат1 должен быть принят не позднее чем через 120 ч, а левоноргестрел и мифепристон — в течение максимум 72 ч после эпизода потери контроля над фертильностью. Следует помнить, что у пациенток с ожирением в качестве экстренной контрацепции предпочтительнее использование улипристала ацетата* в связи с отсутствием влияния повышения индекса массы тела (ИМТ) на эффективность препарата [14]. А в случае выбора девушкой с массой тела более 70 кг или ИМТ выше 26 кг/м² левоноргестрела необходимо увеличить дозу препарата вдвое (до 3,0 мг) [15].

Применение левоноргестрела с целью устранения последствий незащищенного полового акта и выбора гормонального препарата в качестве основного способа контроля фертильности в дальнейшем требует воздержания или дополнительного метода контрацепции как минимум последующие 7 дней.

При отсутствии менструации через 21 день после применения экстренной контрацепции, при «необычности» цикла (меньшие по продолжительности или интенсивности выделения, более болезненные, др.) следует провести повторный тест на беременность [12]. В случае положительного результата подросток должен быть проинформирован об отсутствии свидетельств нежелательного влияния средств экстренной контрацепции на течение беременности и ее исходы [16].

Шаг 2. Акцент на предупреждении инфекций, передающихся половым путем (ИППП), с помощью использования презерватива. Презерватив является единственным на сегодняшний день эффективным средством для предотвращения заражения ИППП, в том числе вирусами иммунодефицита человека и гепатита С. Проблема инфицирования чрезвычайно актуальна как для пациентов с сахарным диабетом, так и для девушек с избыточным весом и ожирением. Дефицит инсулина, неудовлетворительная компенсация нарушений углеводного обмена способствуют развитию вторичного иммунодефицита, хроническая гипергликемия создает благоприятные условия для развития микроорганизмов [17]. Даже незначительный избыток жировой ткани в организме сопровождает хроническое системное воспаление, а длительные нарушения метаболических процессов, гормонального баланса при ожирении изменяют функциональные возможности иммунокомпетентных клеток [18]. Все это повышает риски возникновения инфекции и осложняет процесс элиминации возбудителя.

Однако следует помнить, что в связи с недостаточной эффективностью в плане предотвращения зачатия (индекс Перля 18% при типичном, 2% — при надлежащим применении [3]) рекомендуется использование презерватива в обязательной комбинации с каким-либо другим из контрацептивных средств [9][19].

Шаг 3. При подборе контрацептива — подтверждение выбора письменным информированным согласием. Многими поддерживается мысль о необходимости оформления отдельного правового документа для защиты врача в случае конфликтных ситуаций. Документ должен содержать информацию о выборе подростком или его законным представителем метода контрацепции.

Шаг 4. Принцип безопасности — определение противопоказаний к применению препаратов. Для использования методов контрацепции ВОЗ внедрены Медицинские критерии приемлемости, благодаря которым повышается качество медицинской помощи при планировании семьи. Медицинские критерии приемлемости рассматривают безопасность каждого из методов в отношении здоровья пользователя с двух позиций: 1) создает ли дополнительные риски для здоровья способ контрацепции или осложняет медицинское состояние; 2) влияет ли на эффективность метода контрацепции клиническое состояние [3]. Согласно этим критериям, выделено 4 ситуации (категории Медицинских критерий приемлемости для использования методов контрацепции (Medical Eligibility Criteria, MEC): 1 — отсутствие огрaничений для выбора данного метода контрацепции; 2 — преимущества использования способа контрацепции превалируют над рисками; 3— риски превалируют над преимуществами использования способа контрацепции; 4— применение способа контрацепции представляет собой неприемлемый риск для здоровья.

Вне всяких сомнений, у подростков редко диагностируются заболевания, серьезно ограничивающие выбор контрацептивного метода. Однако сахарный диабет несколько раз упоминается в перечне абсолютных противопоказаний (категории 3/4) к использованию комбинированных гормональных контрацептивов: в случае формирования поздних осложнений (нефропатия/ретинопатия/нейропатия), при длительности заболевания более 20 лет, в сочетании с другими сосудистыми патологиями и в качестве одного из факторов риска сердечно-сосудистых заболеваний (курение, сахарный диабет, артериальная гипертензия). А артериальная гипертензия, часто сопровождающая пациенток с избытком веса или ожирением уже в подростковом возрасте, не позволяет говорить о гормональной контрацепции при систолическом компоненте ≥160 мм рт.ст. и/или диастолическом ≥100 мм рт.ст. [3].

Все это обосновывает необходимость проведения медицинского обследования девушек перед назначением гормональной контрацепции, включающего в себя тщательный сбор личного и семейного анамнеза, оценку здоровья с точки зрения имеющихся проблем в момент обращения, визуальный осмотр с обязательным измерением артериального давления.

Шаг 5. Контрацептивный анамнез. Важно выяснить присутствие у подростка возможных побочных реакций на корригирующие фертильность препараты. Зачастую негативный опыт взаимодействия с контрацептивами обусловлен погрешностью в приеме препарата или относится к так называемым приспособительным реакциям (болевые ощущения в молочных железах, сонливость, изменение/повышение аппетита, лабильность настроения, тошнота, проходящие в течение 3 мес приема препарата).

Побочные же эффекты на фоне применения контрацептивов, как правило, вызваны недостатком или избытком для конкретного организма гормональных влияний.

Наиболее серьезной из побочных реакций применения эстрогенов в составе контрацептивных средств считают венозную тромбоэмболию и тромбоэмболию легочной артерии, которые регистрируются у 7–10/10 000 женщино-лет на фоне приема комбинированных оральных контрацептивов (КОК) и лишь у 4–5/10 000 женщино-лет — у не использующих гормональную контрацепцию. Важно помнить, что вероятность этого осложнения возрастает при наличии других факторов риска тромбоза — курения и врожденных нарушений свертывания крови. Однако к концу 12 мес использования эстрогенсодержащих препаратов, как и через 30 дней после их отмены, риск нарушений в системе свертывания возвращается к исходному [20].

Шаг 6. Осознанный выбор контрацептива. В подростковом возрасте могут быть предложены все методы контрацепции, доступные взрослым пользователям, за исключением стерилизации [9]. Разговор следует начинать с рекомендации наиболее эффективного средства с рассмотрением положительных и отрицательных моментов его применения. Нецелесообразно основой для выбора метода контрацепции делать его стоимость или способ введения. Доказано, что такой подход позволяет наилучшим образом контролировать риски нежелательной беременности [9][12]. Пациентки подросткового возраста с сахарным диабетом длительностью менее 20 лет в отсутствие признаков поздних осложнений имеют возможность применения любого из обратимых методов контрацепции [3][13][19]. В настоящий момент предпочтение в подростковом возрасте отдается применению обратимых контрацептивов пролонгированного действия — Lоng аcting rеvеrsible contraceptives (LARCs) в связи сих высокой эффективностью и безопасностью. Данные средства, не предполагая частых визитов в медицинские учреждения, позволяют сохранить высокий уровень конфиденциальности, не создают проблем в соблюдении режима лечения, обеспечивают быстрое восстановление фертильности после удаления и имеют минимум противопоказаний [21].

К группе LARC в настоящий момент относят инъекции медроксипрогестерона ацетата (кратность применения 1 раз в 3 мес), внутриматочную гормональную систему с левоноргестрелом (эффективность — 5 лет), Cu-ВМС (эффективность — 3–10 лет) и подкожные импланты с этоногестрелом (период действия — 3 года) [22]. Однако назначение депо-препаратов медроксипрогестерона ацетата в подростковом возрасте не приветствуется в связи с их выраженным негативным воздействием на минеральную плотность костной ткани [23]. Информация о влиянии данного средства контрацепции на углеводный обмен (повышение как тощакового, так и постпрандиального уровня гликемии) [15] и массу тела [24] не позволяет говорить об их применении у пациенток с сахарным диабетом и ожирением.

Некоторые авторы причисляют к LARCs еще и комбинированные вагинальные кольца и накожные пластыри [25]. Вагинальные кольца вводятся пользователем самостоятельно на 3 нед, сохраняя эффективность на протяжении 28 дней. Извлеченное преждевременно кольцо может быть возвращено во влагалище не позднее чем через 3 ч. Пластыри помещаются девушкой на ягодицах, предплечьях, верхней части туловища или животе еженедельно в течение 3 нед, 4-я неделя — перерыв виспользовании. Эффективность данных средств серьезно зависит от приверженности пользователя, степени соблюдения им правил применения метода.

В рекомендациях ВОЗ [3] указано, что гестагенсодержащие внутриматочные спирали, импланты и таблетированные формы являются безопасной альтернативой для пациенток, имеющих противопоказания для назначения эстрогенсодержащих контрацептивов, в связи с отсутствием эстрогензависимых влияний на метаболические параметры организма [26]. Подтверждающим безопасность гестагенных методов предохранения от беременности при осложненных формах сахарного диабета и ожирении свойством является отсутствие их негативного влияния на показатели липидного профиля, сывороточной концентрации глюкозы, инсулина и гликированного гемоглобина [22]. Не отмечено снижения минеральной плотности кости на фоне длительного применения этоногестрелсодержащих имплантов [27], что также принципиально для продолжающего свое развитие организма подростка.

Популярность LARCs среди подростков подтверждают результаты образовательного проекта “Choice Project”, в который были вовлечены 1404 девушки 14–19 лет, получившие подробную информацию о различных методах контрацепции — обратимых длительно действующих средствах, комбинированных оральных контрацептивов, вагинальных кольцах и пластырях [28]. Чаще всего предпочтения были отданы внутриматочным системам и имплантам (72%), причем на гестагенсодержащие подкожные импланты указали 34,5% респондентов, 12% подростков выбрали комбинированные оральные контрацептивы. По истечении 2 лет использования сохранение приверженности терапии было также значимо выше в группе применения LARCs, нежели при использовании комбинированных оральных контрацептивов (77 и 44% соответственно) [29].

Но, несмотря на все преимущества обратимой длительно действующей контрацепции, менее 5% девушек используют этот метод в реальной клинической практике [30]. Наиболее значимыми барьерами для использования подростками средств изданной группы являются необходимость участия специалиста-гинеколога в процедуре введения системы и ее цена [31]. Но простой учет срока эффективного применения метода доказывает сравнительно меньшую его стоимость по сравнению сосредствами, требующими ежедневного использования [32].

Высокая эффективность подкожных имплантов, содержащих этоногестрел, и внутриматочных систем достигается главным образом за счет уменьшения количества цервикальной слизи, повышения ее вязкости и предотвращения проникновения сперматозоидов в полость матки, однако есть сообщения и о возможности подавления ими овуляции. Cu-ВМС, вызывают воспалительную реакцию при высвобождении этого элемента в полость матки — ионы меди, простагландины и макрофаги создают токсичную для спермы и ооцитов среду [21]. Дополнительным преимуществом Cu-ВМС является возможность их применения в качестве средства экстренной контрацепции после незащищенного полового акта [3]. O’Brien S. и соавт. (2017), оценивая риски тромбоэмболических осложнений у женщин с сахарным диабетом 1 и 2 типов, заключили, что внутриматочные системы и подкожные гестагенсодержащие импланты в меньшей, чем комбинированные оральные контрацептивы, степени связаны с этими серьезными состояниями [33].

К сожалению, сравнительных исследований применения различных способов контрацепции у пациенток с сахарным диабетом, и особенно — подросткового возраста, предоставляющих достаточную доказательную базу, крайне мало. Тем не менее совершенно очевидно, что при выборе метода контроля фертильности при нарушении углеводного обмена следует учитывать такие параметры, как длительность основного заболевания, качество гликемического контроля, наличие поздних специфических осложнений, ожирения, артериальной гипертензии [3].

Метаанализ, проведенный Gariani K. и соавт., доказал повышение риска венозных тромбоэмболий у пациентов с сахарным диабетом как 1, так и 2 типов [34]. Поэтому для девушек с патологией углеводного обмена нежелателен дополнительный протромботический эффект эстрогенсодержащих контрацептивных препаратов. В случае наличия у пациентки специфических осложнений сахарного диабета альтернативой комбинированным гормональным контрацептивам может стать Cu-ВМС (категория МЕС 1), левоноргестрелсодержащая ВМС или гестагенсодержащий имплант (категория МЕС 2) [3][21].

Очень важно при определении метода контрацепции оценить приверженность девушки рекомендациям по метаболическому контролю основного заболевания. В случае низкого комплаенса назначение средств, требующих применения с достаточно высокой кратностью (например, оральные контрацептивы), не позволит добиться необходимой эффективности и может привести к незапланированной беременности на фоне неудовлетворительных метаболических параметров (гипергликемия, дислипидемия) [21].

Принципиальным моментом при выборе способа коррекции фертильности у пациенток с сахарным диабетом и ожирением является оценка риска его влияния на метаболизм. Последствия применения КОК, содержащих эстроген, во многом определяются его дозой. Однозначно, что популярные ранее высокие дозы этинилэстрадиола связаны с прогрессированием поздних специфических осложнений сахарного диабета [19]. Однако есть сведения и о возможности превентивного действия низкодозированных КОК в отношении развития макроангиопатий [35].

Diab К. и соавт. (2000), оценивая назначение женщинам с удовлетворительной компенсацией сахарного диабета (HbA1c менее 8%) Cu-ВМС, КОК и депо-препаратов медроксипрогестерона ацетата, отметили некоторое повышение уровня гликемии натощак без значимого изменения дозы инсулина при использовании препаратов двух последних групп [36].

Более поздние работы свидетельствуют о значимом ухудшении кардиоваскулярного профиля у пациенток подросткового возраста с сахарным диабетом 1 типа на фоне приема комбинированных оральных контрацептивов за счет негативных изменений липидного спектра крови и повышения артериального давления [37].

Влияние подкожных гестагенсодержащих имплантов на метаболический контроль при сахарном диабете было оценено в двух исследованиях — ни в одном из них не отмечено дестабилизации гликемии, изменения потребности в инсулине, влиянияна ИМТ или уровень HbA1c, зарегистрировано улучшение липидного спектра крови и уменьшение альбуминурии [21][36]. Также отсутствуют свидетельства отрицательного влияния на гликемию, уровень HbA1c, потребность в сахароснижающей терапиии липидный профиль у женщин с сахарным диабетом 1 и 2 типов при длительном использовании ВМС с левоноргестрелом. Риск выпадения и местных воспалительных реакций при длительном использовании данных систем на фоне сахарного диабета составил 3,7 и 1,7% соответственно [38], не отличался от таковых у пользователей без диабета и был сравним у рожавших и нерожавших женщин [39].

Выбор контрацепции для девушки с ожирением имеет дополнительные нюансы. Faculty of Sexual & Reproductive Healthcare (FSRH) рекомендует при обсуждении методов коррекции фертильности при наличии высокого ИМТ у пациентки отвечать наследующие вопросы:

На сегодняшний день имеются убедительные доказательства одинаково высокой эффективности Cu-ВМС и левоноргестрела как у женщин с нормальной массой тела, так и у пользователей с избытком веса и ожирением [40][41]. Cu-ВМС не имеют ограничений в использовании с позиций безопасности контрацепции [3]. Метаанализ результатов 8 наблюдательных исследований не выявил связи применения гестагенсодержащих ВМС на риски венозных тромбоэмболий, однако необходимо заметить, что исследование проведено без учета ИМТ женщин [42]. Отсутствуют доказательства набора веса на фоне применения какого-либо из вариантов ВМС [15]. Дополнительной выгодой от применения данного вида контрацепции может стать снижение рисков гиперплазии и рака эндометрия, значимо повышенных у пациенток с ожирением [43].

Несмотря на информацию об изменении фармакокинетики этоногестрела, содержащегося в подкожных имплантах, на 4–5-м годах использования (то есть за рамками рекомендованного срока) у некоторых женщин с ИМТ более 25 кг/м² наблюдается снижение его концентрации в крови ниже 90 пг/мл. Данная ситуация не имеет клинического значения, поскольку ни в одном из исследований риск нежелательной беременности у женщины с избытком массы тела или ожирением не отличался от такового у пользователей с нормальным ИМТ [44]. В связи с отсутствием свидетельств влияния имплантов и таблетированных форм, содержащих гестаген, на кардиоваскулярные риски увеличение таких событий, как инфаркт миокарда, инсульт ивенозные тромбоэмболии, этот способ коррекции фертильности считается безопасным, в том числе и для пациенток с избыточной массой тела и ожирением [42]. При анализе влияния гестагенсодержащих имплантов и оральных форм контрацепции навсю популяцию пользователей в целом не было получено убедительных доказательств их негативного влияния на массу тела при исходном повышении ИМТ. Неконтрацептивными эффектами при использовании данных средств может стать облегчение ситуации в плане дисменореи и болевого синдрома при овуляции, если эти отклонения не связаны с серьезной органической патологией [15].

Сведения о рисках незапланированной беременности при использовании КОК у женщин с избытком веса и ожирением разноречивы. Имеются доказательства неизменной эффективности КОК при повышенном ИМТ [45]. Сообщается также и о повышении овариальной активности во время 7-дневного перерыва в приеме препаратов [46]. Однако Кохрейновский обзор (2016) сделал заключение об отсутствии зависимости действия КОК от веса женщины [41].

Согласно результатам нескольких исследований, при весе женщины более 90 кг снижается эффективность применения комбинированных трансдермальных пластырей [47], о некотором повышении риска незапланированной беременности на фоне использования вагинального кольца говорят уже при массе тела от 75 до 84 кг [48].

Параллельно с увеличением ИМТ повышается возможность развития тромбозов глубоких вен [49], поэтому назначение любой из форм комбинированных гормональных контрацептивов, содержащих эстрогены, при ИМТ выше 35 кг/м² небезопасно [50]. Систематический анализ свидетельствует об увеличении распространенности инфарктов миокарда и инсультов у пациенток с повышением ИМТ на фоне приема КОК [51]. Достоверных данных о влиянии комбинированных контрацептивов на массу тела пользователей не зарегистрировано [15]. Дополнительными преимуществами при использовании комбинированных гормональных контрацептивов станут облегчение предменструального синдрома, коррекция симптомов, связанных споликистозом яичников, и снижение рисков развития рака яичников, эндометрия и колоректального рака [52].

Шаг 7. Выбор режима применения контрацептивов. Необходимо выяснить предпочтения подростка по режиму применения и способу введения препарата в организм. Для этого информация о приемлемом для него спектре контрацептивных средств должна быть предоставлена ему в доступной для понимания форме. Одним из методов повышения приверженности выполнению рекомендаций по использованию контрацепции считают немедленный старт приема выбранного препарата — «Quick Start», отказ от необходимости дожидаться очередной менструации [53]. После исключения возможности беременности при отсутствии противопоказаний можно начать прием комбинированных гормональных контрацептивов (оральные формы, вагинальное кольцо, пластыри), инъекционных гестагенов, контрацептивных имплантов и ВМС. Но подросток может отложить старт контрацепции до начала следующего менструального цикла, дабы окончательно убедиться в отсутствии беременности, или до ближайшего воскресенья в случае применения комбинированных гормональных средств (“Sunday start”) для планирования кровотечения отмены [12].

При отсутствии жалоб и симптомов нет необходимости в проведении гинекологического осмотра при старте контрацепции, скрининг на ИППП также необязателен перед введением ВМС, может быть проведен в день установки системы, нет необходимости откладывать процедуру введения до получения результатов в случае если нет явных признаков инфекции. У девушек с ожирением введение ВМС может иметь дополнительные сложности в плане определения положения матки [54], однако повышение ИМТ не является значимым фактором снижения эффективности этого метода [40], и трудности при установке легко устраняются опытным специалистом.

Cu-ВМС эффективна с момента установки, а комбинированные гормональные контрацептивы, ВМС с левоноргестрелом, гестагеновые импланты и медроксипрогестерона ацетат обеспечивают контрацепцию через 7 дней после старта. В случае выбора последних в первые 7 дней необходимо применение добавочных способов контрацепции (например, барьерных) или воздержание от интимной близости, а через 2–4 нед должен быть проведен тест на беременность [12].

Шаг 8. Разъяснение правил применения методов предохранения, побочных эффектов и возможных неблагоприятных клинических реакций, особенностей наблюдения пациентки. После завершения процедуры осознанного выбора необходимо обсудить с подростком вопросы дальнейшего взаимодействия.

Некоторое повышение риска местных гинекологических воспалительных заболеваний в течение 21 дня после введения ВМС обосновывает необходимость скрининга на ИППП в день введения и терапии в случае положительного результата ссохранением установленной системы [55]. Подросток должен быть проинформирован о наиболее частых побочных эффектах, возникающих в первые 3–6 мес использования ВМС, гестагенсодержащих имплантов и таблетированных средств, — нерегулярных кровотечениях [56]. Применение левоноргестрелсодержащих ВМС достаточно часто приводит к появлению акне (25% пользователей), головной боли (14%) и перепадам настроения (11%) [57]. Серьезное отношение к количеству потребляемых калорий обосновано сведениями о наборе веса в 5–22% случаев на фоне использования гестагенсодержащих контрацептивов [58].

В связи с невозможностью однозначного исключения влияния комбинированных гормональных контрацептивов на гомеостаз глюкозы, кардиоваскулярные риски (липидный профиль и артериальное давление) и венозные тромбоэмболии [59] девушек необходимо уведомить о необходимости визита к врачу через 3 мес после начала приема препарата и затем ежегодно. Надлежащими являются проведение гинекологического обследования, включающего кольпоскопию и цитологическое исследование, регулярное измерение артериального давления, оценка липидного спектра крови и осмотр молочных желез [8].

## ЗАКЛЮЧЕНИЕ

В подростковом возрасте могут быть предложены все методы контрацепции, доступные взрослым пользователям, за исключением стерилизации, однако рекомендуется комбинация барьерного метода (презерватив) и какого-либо другого средства для профилактики как ИППП, так и нежелательной беременности. Наиболее приоритетным принципом при выборе метода контрацепции у подростка является оценка его эффективности. Пациентки подросткового возраста с сахарным диабетом длительностью менее 20 лет в отсутствие признаков поздних осложнений имеют возможность применения любого из обратимых методов контрацепции. Назначение комбинированных гормональных контрацептивов (КОК, пластыри, вагинальные кольца) нежелательно у девушек при наличии поздних осложнений сахарного диабета и с ожирением при ИМТ более 35 кг/м². Гестагеновые контрацептивы (внутриматочные системы, импланты и таблетированные формы) и Cu-ВМС являются безопасной альтернативой для девушек, имеющих противопоказания для назначения эстрогенсодержащих контрацептивов (поздние специфические осложнения сахарного диабета, ожирение с ИМТ более 35 кг/м²). Применение контрацептивов — показание для динамического наблюдения за девушкой для предупреждения, своевременного выявления и коррекции нежелательных последствий.

## ДОПОЛНИТЕЛЬНАЯ ИНФОРМАЦИЯ

Источники финансирования. Работа выполнена по инициативе авторов без привлечения финансирования.

Конфликт интересов. Авторы декларируют отсутствие явных и потенциальных конфликтов интересов, связанных с содержанием настоящей статьи.

Участие авторов. Шайдуллина М.Р — дизайн исследования, анализ данных, написание статьи, редакционная правка; Валеева Ф.В — концепция исследования, анализ данных, написание статьи, редакционная правка; Субханкулова А.Ф — концепция исследования, анализ данных, редакционная правка; Хусиева П.А — дизайн исследования, анализ литературы, редакционная правка. Все авторы одобрили финальную версию статьи перед публикацией, выразили согласие нести ответственность за все аспекты работы, подразумевающую надлежащее изучение и решение вопросов, связанных с точностью или добросовестностью любой части работы.

1. улипристала ацетат — синтетический селективный модулятор прогестероновых рецепторов, не зарегистрирован на территории РФ.
